# Echocardiographic and Hemodynamic Effects of Intraaortic Balloon Pump in Patients with Cardiogenic Shock on Veno-Arterial Extracorporeal Membrane Oxygenation

**DOI:** 10.3390/jcm14113687

**Published:** 2025-05-24

**Authors:** Misa Fister, Tomaz Goslar, Peter Radsel, Marko Noc

**Affiliations:** 1 Clinical Department of Intensive Internal Medicine, University Medical Center Ljubljana, Zaloska 7, 1000 Ljubljana, Slovenia; tomaz.goslar@kclj.si (T.G.); peter.radsel@kclj.si (P.R.); marko.noc@mf.uni-lj.si (M.N.); 2 Medical Faculty, University of Ljubljana, Korytkova ulica 2, 1000 Ljubljana, Slovenia

**Keywords:** cardiogenic shock, intraaortic balloon pump, VA ECMO

## Abstract

We investigated echocardiographic and hemodynamic effects of intraaortic balloon pump (IABP) in 26 patients with cardiogenic shock on veno-arterial membrane oxygenation (VA ECMO). Our study demonstrated an 8.1% increase in left ventricular velocity time integral (*p* = 0.023) without reduction in left ventricular diameters and 4.7% decrease in right ventricular end diastolic base diameter (*p* = 0.05) when using IABP 1:1 mode compared to no augmentation. This was associated with a 3.2% decrease in heart rate (*p* < 0.001) and a 3.0% increase in mixed venous oxygen saturation (*p* = 0.057). Since the magnitude of the documented favorable changes is rather small, the clinical relevance of concomitant IABP in patients with cardiogenic shock on VA ECMO remains questionable.

## 1. Introduction

Patients with cardiogenic shock are increasingly treated with temporary mechanical circulatory support devices, which serve as a bridge to recovery, a long-term assist device, or heart transplantation. Veno-arterial extracorporeal membrane oxygenation (VA ECMO), usually implanted in the catheterization laboratory by a skilled interventional cardiologist, is frequently used to support both failing left and right ventricles [1]. Unfortunately, retrograde ECMO flow may increase aortic pressure and thereby left ventricular afterload [2,3]. This may result in ventricular distension and blood stasis, leading to thrombus formation, systemic embolization, and impaired myocardial recovery. Furthermore, elevated left ventricular pressure may also reduce subendocardial perfusion and intensify myocardial ischemia [4].

There are several medical and surgical options to decrease left ventricular afterload [2,5]. The first step is usually the so-called “partial flow strategy”, aimed to provide adequate end-organ perfusion with the least possible retrograde ECMO flow [6]. Additionally, the least invasive mechanical option, which may reduce left ventricular afterload and thereby improve the opening of the aortic valve, is the intraaortic balloon pump (IABP) [7].

Bedside echocardiography, which should be available on a 24/7 basis, represents an essential imaging tool to guide left ventricular unloading strategies in patients on VA ECMO [8]. Since patients are usually in a supine position, it is sometimes challenging to obtain adequate acoustic windows. However, the velocity time integral of left ventricular outflow tract (VTI LVOT), which may serve as a simple quantitative index of left ventricular stroke volume, can usually be obtained [9]. Furthermore, a progressive increase in left ventricular dimensions despite the “partial flow strategy” represents another valuable echocardiographic parameter arguing for the initiation of unloading strategies [10]. Moreover, echocardiography is also very valuable during VA ECMO discontinuation because VTI LVOT below 10 cm predicts failure of weaning [11].

In the present study, we focused on patients with cardiogenic shock supported by VA ECMO and aimed to quantify the echocardiographic and hemodynamic effects of concomitant IABP.

## 2. Methods

This was a single-center, prospective, nonrandomized study in patients with cardiogenic shock on VA ECMO and concomitant IABP conducted at the Center for Intensive Internal Medicine of the University Medical Center Ljubljana (Slovenia). The study was approved by the National Ethics Committee (Number 0120-377/2020/6, 17 November 2020).

**Figure 1:**
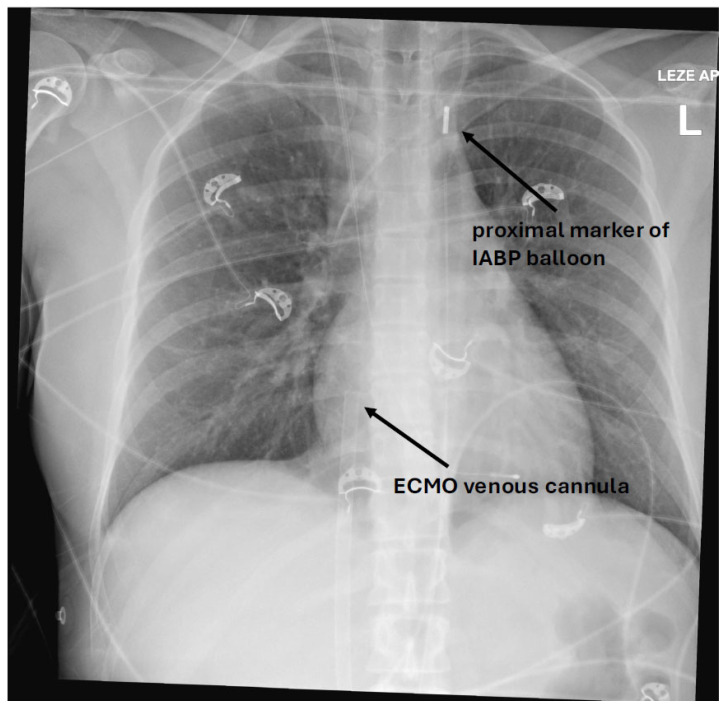
X-ray of a patient showing a proximal IABP balloon marker in aortic knob and an ECMO venous cannula with the tip in the right atrium.

Patients were in a supine position, sedated, intubated on mechanical ventilation, and on VA ECMO (Cardiohelp, Maquet Getinge, Rastatt, Germany) with concomitant IABP (Cardiosave IABP, Maquet Getinge, Rastatt, Germany). We used the largest fiberoptic balloon according to patient height (Figure 1). Patients with inadequate echocardiographic windows and patients hospitalized for less than 24 h were excluded from the study.

**Figure 2:**
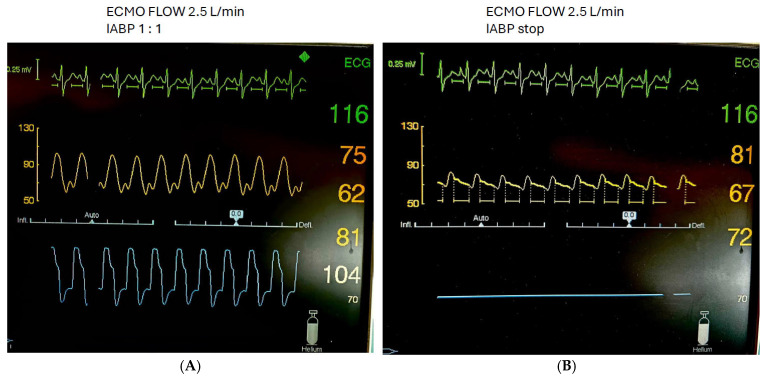
Patient with cardiogenic shock on VA ECMO and concomitant IABP with 1:1. Mode (**A**) and IABP stop (**B**).

Measurements, performed by a single investigator skilled in echocardiography, were initially obtained on 1:1 IABP augmentation mode. IABP support was then gradually decreased in increments of 10 min to 1:2 and 1:3, followed by a complete IABP stop for 10 min. After reaching a stable hemodynamic condition at the complete stop, the measurements were repeated (Figure 2). VA ECMO flow, sweep gas, mechanical ventilatory support, and vasopressor/inotropic therapy remained constant during the measurements. Only norepinephrine and dobutamine were used in this patient cohort. The degree of vasopressor/inotropic support was expressed as catecholamine index.

The following parameters were collected:

- Echocardiographic measurements: VTI LVOT (in cm, average of 3 cardiac cycles), left ventricular end diastolic diameter (LVEDD), left ventricular end systolic diameter (LVESD), and right ventricular end diastolic base dimension (RVEDD);
- Hemodynamic measurements: heart rate, arterial pressure, mixed venous oxygen saturation (SvO_2_) from venous blood before VA ECMO oxygenator, and end tidal carbon dioxide tension (etCO_2_).

Numerical data are shown as mean ± standard deviation, and categorical data as numbers and proportions in percentages. Differences in numerical measurements between IABP 1:1 and IABP stop were assessed by a paired t-test. A *p*-value of <0.05 was considered significant.

## 3. Results

Between 2014 and 2021, among 119 consecutive patients with cardiogenic shock on VA ECMO, 60 patients (50%) had concomitant IABP. Since 15 patients were hospitalized for less than 24 h and 19 patients had suboptimal acoustic windows or the investigator/echocardiographist was not available, 26 patients were ultimately enrolled in the study. Because a small number of patients was enrolled, only one imaging modality was used. Echocardiography was performed after 24 h of hospitalization when the patients were more hemodynamically stable. Patients were predominantly men (81%), with a mean age of 53 years, different etiology of cardiogenic shock and a mean arterial lactate of 7.4 mmol/L (Table 1). The patients with acute coronary syndrome were triaged directly in the catheterization laboratory where coronary angiography and percutaneous revascularization were performed along with VA ECMO and IABP implantation. The mean VA ECMO flow during the measurements was 2.1 L/min. In total, 84.5% of patients were weaned off ECMO, and 46% survived to hospital discharge.

**Figure 3:**
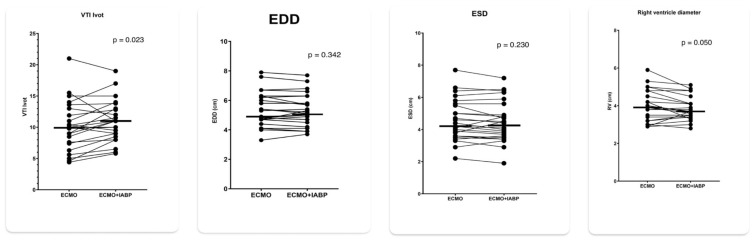
Echocardiographic parameters with 1:1 IABP augmentation and IABP stop in patients on VA ECMO for cardiogenic shock. LV VTI = left ventricular outflow velocity time integral; LVEDD = left ventricular end diastolic diameter; LVESD = left ventricular end systolic diameter: RVEDD = right ventricular end diastolic base diameter.

**Figure 4:**
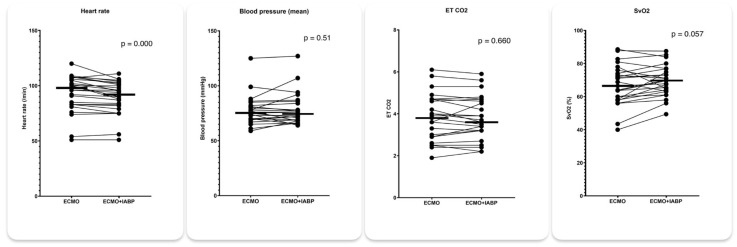
Hemodynamic parameters with 1:1 IABP augmentation and IABP stop in patients on VA ECMO for cardiogenic shock.

IABP mode 1:1 was associated with a significant increase in LVOT VTI from 9.9 to 10.7 (*p* = 0.023) and a decrease in RVEDD from 4.01 to 3.83 (*p* = 0.050) while LVEDD and LVESD remained essentially unchanged (Figure 3). Concomitantly, heart rate decreased from 93 to 90/min (*p* < 0.001) and SvO_2_ increased from 67% to 69% (*p* = 0.057). There was no significant difference in mean arterial pressure and etCO_2_ between 1:1 augmentation and IABP stop (Figure 4).

## 4. Discussion

The main finding of our study was a significant increase in VTI LVOT with 1:1 augmentation compared to IABP stop, indicating an up to 10% increase in left ventricular stroke volume. LVEDD and LVESD, on the other hand, remained essentially unchanged, indicating the inability of the IABP strategy to significantly decrease left ventricular distension. Our finding is in accordance with a closed-loop, real-time computer model of the human cardiovascular system, which demonstrated an increase in left ventricular stroke volume by 5–10% with unchanged pulmonary capillary wedge pressure and LVEDD with concomitant IABP support [12]. We further documented approximately 7% decrease in RVEDD with the 1:1 IABP mode. Since we do not have a sound physiological explanation, this finding is most likely due to chance, due to the small sample size. On the other hand, the unloading of LV may increase native cardiac output and thereby unload the RV. Because of the thinner right ventricular wall and better compliance, RVEDD would, in contrast to LVEDD, decrease.

Favorable echocardiographic changes with 1:1 IABP augmentation were associated with a small but significant decrease in the heart rate and a statistically borderline increase in SvO2, while systemic arterial pressure and etCO_2_ remained essentially unchanged. This is consistent with improved end-organ perfusion with the 1:1 IABP augmentation, despite comparable forward aortic flow, which was roughly estimated by etCO2 in the setting of low transpulmonary blood flow and constant mechanical ventilation. A possible explanation for improved end-organ perfusion in the 1:1 IABP mode may therefore lie in diastolic pressure augmentation during balloon inflation. Improved end-organ perfusion may in part also explain the decreased mortality in patients on VA ECMO and concomitant IABP demonstrated in recent large retrospective meta-analyses [13,14]. Importantly, all favorable echocardiographic and hemodynamic changes documented in our study were rather small and did not exceed 10%. Despite statistical significance, their clinical relevance at the bedside therefore remained questionable. It appears that there are subgroups of patients that may benefit more from IABP implantation. We can speculate that IABP is more efficient in patients without vasodilatation (e.g., concomitant systemic inflammation response syndrome or septic shock) but our study was too small to further address this topic in more detail.

Our study investigated only the echocardiographic and hemodynamic impact of IABP in the setting of cardiogenic shock supported by VA ECMO. We did not address abdominal visceral organ perfusion provided by arterial branches, originating from the descending aorta, which may be covered by an aortic balloon. It has been shown that despite correct aortic balloon position on radiography, computed tomography revealed the compromise of at least one visceral artery, including celiac trunk, superior mesenteric artery, or renal arteries, in 61 of 63 patients [15]. This occurred due to anatomic-to-balloon length mismatch and was associated with mesenteric ischemia and the need for laparotomy in 23.8%. In patients with VA ECMO, visceral hemodynamics may be further complicated due to combined native anterograde pulsative flow and retrograde non-pulsative flow in the descending aorta.

Our study has several important limitations. It was a single-center study, with a rather small number of enrolled patients. We focused only on selected echocardiographic and hemodynamic parameters without addressing end-organ perfusion and especially the perfusion of abdominal visceral organs. The echocardiographist was not blinded to the IABP augmentation and might have been biased toward better measurements in the 1:1 IABP group. Since all patients were on rather low retrograde VA ECMO flow (2.1 L/min), it is likely that with increased flow, the herein described favorable echocardiographic and hemodynamic changes would become even smaller. Moreover, the small sample size did not allow for the identification of subgroups of patients most likely to benefit from a concomitant IABP strategy. Last but not least, because of the small sample size, we could not address the balance between favorable echocardiographic and hemodynamic effects and adverse events related to the concomitant IABP.

## 5. Conclusions

Our study demonstrated an 8.1% increase in the left ventricular velocity time integral without reduction in left ventricular diameter and a 4.7% decrease in the right ventricular end diastolic base diameter when using the IABP 1:1 mode compared to no augmentation. This was associated with a 3.2% decrease in heart rate and a 3.0% increase in mixed venous oxygen saturation. Since the magnitude of the observed changes is rather small, the clinical impact of a concomitant IABP strategy in patients with cardiogenic shock on VA ECMO remains questionable. IABP should therefore be applied selectively in patients where LV unloading with partial flow ECMO support is not sufficient.

## Figures and Tables

**Table 1:** Patient characteristics.

Patients	n = 26
Age, years	53 ± 12
Men	21 (81%)
Body mass index, kg/m^2^	28.1 ± 3.9
Etiology of cardiogenic shock	Acute coronary syndrome 15 (57%) Non-ischemic cardiomyopathy 3 (12%) Acute myocarditis 3 (12%) Other 5 (19%)
ECMO flow, L/min	2.1 ± 2.1
ECMO flow/BSA, L/min/m^2^	1.04 ± 0.54
Catecholamine index *	72.8 ± 49.3
SOFA	11.2 ± 3.5
APACHE 2	22.8 ±7.9
SAPS 2	61.4 ±17.5
Arterial lactate, mmol/L	7.4 ± 5.1
Decannulation survival	22 (84.5%)
Hospital discharge survival	12 (46%)

Legend: APACHE 2 = Acute Physiology and Chronic Health Evaluation II; SOFA = The Sequential Organ Failure Assessment; SAPS 2 = The Simplified Acute Physiology Score II. BSA was calculated using the Du Bois formula. * Catecholamine index was calculated by dopamine dose (μg/kg/min) + noradrenalin and adrenaline dose (μg/kg/min) × 100.

## Data Availability

The original contributions presented in this study are included in the article. Further inquiries can be directed to the corresponding author(s).
